# Imaging Features of Periosteal Chondroma Manifesting as a Subcutaneous Mass in the Index Finger

**DOI:** 10.1155/2014/763480

**Published:** 2014-02-23

**Authors:** Hidetomo Kosaka, Jun Nishio, Taiki Matsunaga, Mikiko Aoki, Hiroshi Iwasaki, Masatoshi Naito

**Affiliations:** ^1^Department of Orthopaedic Surgery, Faculty of Medicine, Fukuoka University, 7-45-1 Nanakuma, Jonan-ku, Fukuoka 814-0180, Japan; ^2^Department of Pathology, Faculty of Medicine, Fukuoka University, 7-45-1 Nanakuma, Jonan-ku, Fukuoka 814-0180, Japan

## Abstract

Periosteal chondroma is a rare benign hyaline cartilage neoplasm that occurs most commonly in the metaphases of long tubular bones. We present a unique case of periosteal chondroma arising in the proximal phalanx of the left index finger in a 12-year-old boy. Physical examination revealed a slightly protuberant, subcutaneous mass. Plain radiographs and computed tomography scans showed a periosteal lesion producing saucerization of the cortex and subjacent cortical sclerosis, without internal matrix calcification. On magnetic resonance imaging, the lesion exhibited intermediate signal intensity on T1-weighted images and high signal intensity on T2-weighted images. Contrast-enhanced fat-suppressed T1-weighted images demonstrated peripheral and septal enhancement. The patient underwent a marginal excision with curettage of the underlying bone cortex. Histological examination confirmed the diagnosis of periosteal chondroma. There has been no evidence of local recurrence eight months after surgery. Periosteal chondroma can protrude into the subcutaneous soft tissue causing a palpable mass. Recognition of the typical radiological features can lead to an accurate diagnosis of this rare condition.

## 1. Introduction

Periosteal chondroma, also known as juxtacortical chondroma, is a rare benign cartilaginous neoplasm of bone surface that occurs predominantly in the metaphases of long tubular bones, particularly, the proximal humerus and distal femur. The small tubular bones are also common sites. It presents as a swelling or palpable mass that may be painful in children and young adults, with a slight male predominance [1]. Histologically, periosteal chondroma consists of lobulated hyaline cartilage with variable cellularity. The chondrocytes frequently show mild nuclear atypia with hyperchromasia and binucleation [2], mimicking chondrosarcoma. Surgical excision is the treatment of choice. Malignant transformation has not been reported. We herein present the imaging features of periosteal chondroma involving the proximal phalanx of the left index finger in an adolescent boy and provide a brief review of the relevant literature.

## 2. Case Presentation

**Figure 1:**
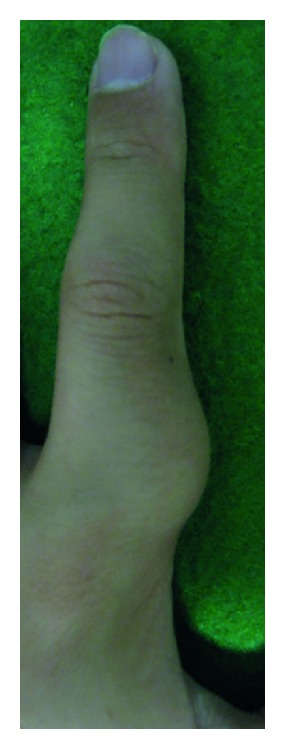
Photograph shows a slightly protuberant, subcutaneous mass in the radial aspect of the proximal phalanx of the left index finger.

A 12-year-old boy presented with a 1-year history of a slow-growing, painless mass in the radial aspect of the proximal phalanx of the left index finger. The patient's past medical history was unremarkable. Physical examination revealed a 2 cm, firm, immobile, nontender, subcutaneous mass (Figure 1). There was no limitation of motion in neighboring joints. Neurovascular examinations were normal. Laboratory data were within normal limits.

**Figure 2:**
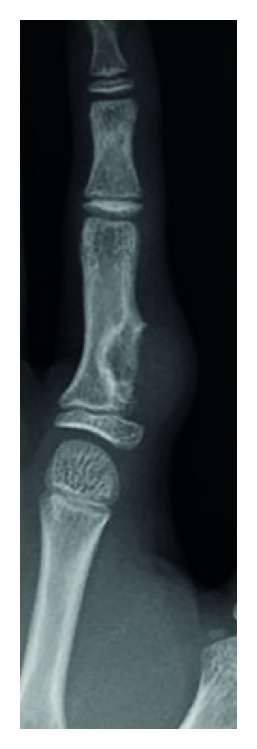
Anteroposterior radiograph reveals saucerization of the underlying cortex and a rim of sclerosis on the radial surface of the proximal phalanx.

**Figure 3:**
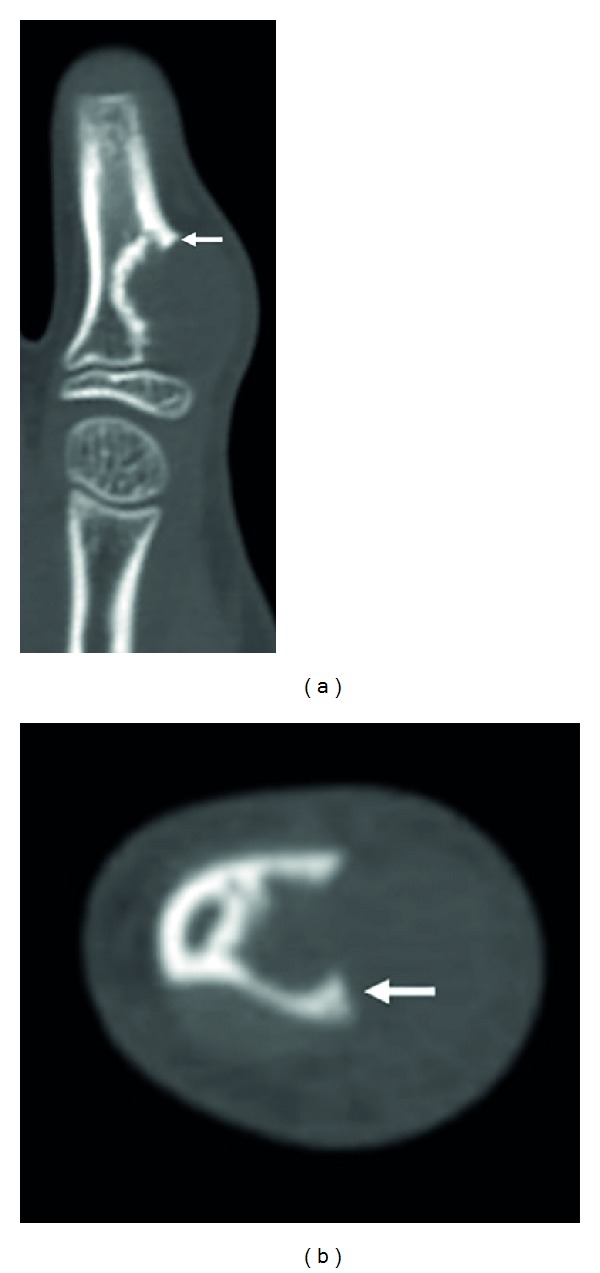
Coronal (a) and axial (b) computed tomography scans show cortical overhanging margins at the periphery of the lesion (arrows).

**Figure 4:**
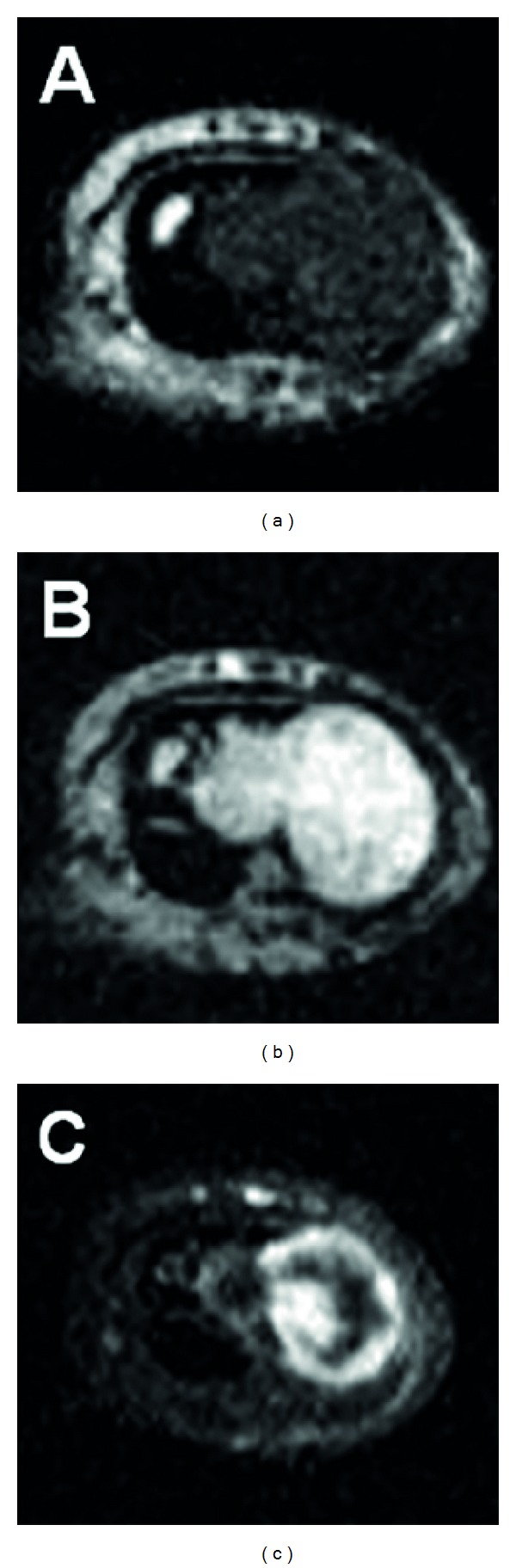
Axial magnetic resonance imaging showing a well-defined juxtacortical mass with intermediate signal intensity on T1-weighted image (a) and high signal intensity on T2-weighted image (b). Contrast-enhanced fat-suppressed T1-weighted image (c) demonstrates peripheral and septal enhancement.

Plain radiographs revealed a discernible soft tissue lesion with saucerization and sclerosis of the underlying cortex (Figure 2). Computed tomography (CT) scans confirmed the presence of a periosteal-based lesion without calcification (Figure 3). Magnetic resonance imaging (MRI) exhibited a well-circumscribed juxtacortical mass measuring 1.8 × 1.2 × 0.8 cm. The mass showed intermediate signal intensity on T1-weighted images (Figure 4(a)) and high signal intensity on T2-weighted images (Figure 4(b)). Contrast-enhanced fat-suppressed T1-weighted images demonstrated peripheral and septal enhancement (Figure 4(c)). Based on these findings, periosteal chondroma was considered.

**Figure 5:**
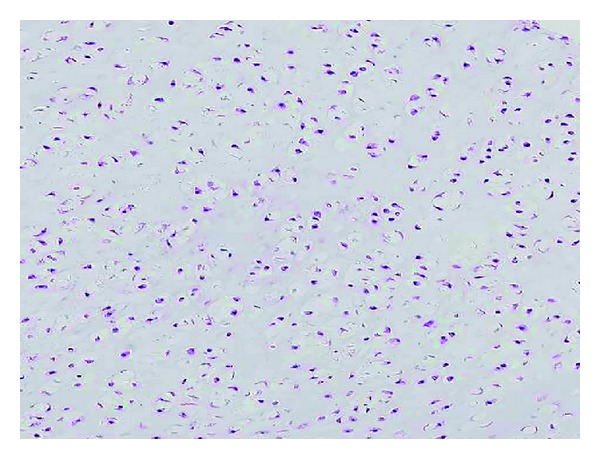
Histological finding of periosteal chondroma. The tumor is composed of bland chondrocytes in an abundant hyaline cartilage matrix.

The operative procedure was carried out under general anesthesia with tourniquet control. A zigzag incision was made on the volar aspect of the left index finger. The mass was adherent to the periosteum of the proximal phalanx and extended into the bone cortex. It was completely excised together with the overlying peritoneum, and the underlying bone cortex was curetted. There was no sign of invasion into the medullar cavity. Bone graft was not done. Histologically, the tumor was well demarcated and surrounded by a periosteum-like fibrous capsule. The tumor consisted of hyaline cartilage with clusters of bland chondrocytes (Figure 5). Binucleated cells were occasionally observed, but mitotic activity was absent. The histological findings were compatible with periosteal chondroma.

The postoperative course was uneventful, and the patient is doing well without evidence of local recurrence eight months after surgery.

## 3. Discussion

Surface-based bone tumors may present as palpable subcutaneous masses in the hands or feet, as in our case. In fact, periosteal chondromas involving finger phalanges were confused with primary subcutaneous or skin tumors at initial presentation [3, 4]. Clinicians should therefore be aware of the possibility of periosteal chondroma when evaluating subcutaneous lesions in the fingers.

Periosteal chondroma is much less common than enchondroma, accounting for less than 2% of all chondromas [1]. It is usually small, measuring less than 3 cm in diameter, and can erode the underlying cortex without penetrating into the medullar cavity. Patients with periosteal chondromas of the fingers are usually asymptomatic and they complain only focal swelling or palpable masses [3, 4]. The pathogenesis of periosteal chondroma is poorly understood, but *isocitrate dehydrogenase 1* (*IDH1*) mutations have been identified in 71% of cases [5]. Histologically, the lesions occasionally show hypercellularity, nuclear enlargement, binucleation, and myxoid change of the matrix, which can lead to a mistaken diagnosis of low-grade chondrosarcoma [2]. It is therefore important to be familiar with the imaging features of periosteal chondroma for its accurate diagnosis and appropriate treatment.

Plain radiographs often show a discernible soft tissue mass with underlying cortical saucerization or scalloping, subjacent cortical sclerosis, and overhanging margins [6]. CT may be helpful in identifying the presence of scattered calcification and the lack of intramedullary extension. On MRI, periosteal chondroma typically appears as a well-circumscribed, juxtacortical mass with intermediate signal intensity on T1-weighted images and high signal intensity on T2-weighted images [7]. Extraosseous soft tissue edema adjacent to the lesion may be seen on T2-weighted images [6]. Periosteal chondroma usually demonstrates peripheral enhancement after intravenous gadolinium administration [7]. The imaging findings in our case were consistent with the above-mentioned findings.

Differential diagnosis of the current case includes soft tissue chondroma and periosteal chondrosarcoma. Soft tissue chondroma occurs most commonly in the fingers and typically affects adults between 30 and 60 years of age [8]. Radiographically, the lesion is well demarcated and does not involve the underlying bone, although some tumors cause compression deformity or bone erosion [9]. Histologically, soft tissue chondroma and periosteal chondroma are similar, and the distinction is best made on the basis of radiological features. Periosteal chondrosarcoma occurs predominantly in the metaphases of long tubular bones and has a peak incidence in the second to fourth decades of life [10]. The small tubular bones are rarely affected. The lesion is generally larger with a more prominent soft tissue component. According to Robinson et al. [11], lesion size is the most reliable predictor to differentiate between these two neoplasms. However, permeation into the soft tissue is an important characteristic of periosteal chondrosarcoma that can be used to distinguish it from periosteal chondroma [10].

Periosteal chondroma is usually treated with surgical excision. Local recurrence is extremely uncommon [1] and is associated with incomplete excision. Resection of the underlying bone cortex after excision of this tumor has been suggested to reduce the possibility of recurrence [12].

In summary, we have described a unique case of periosteal chondroma of the proximal phalanx in the left index finger manifesting as a subcutaneous mass in an adolescent boy. Knowledge of the characteristic imaging features can lead to an accurate diagnosis of periosteal chondroma, thereby avoiding unnecessary radical surgery.
